# Supplementary material for: Are carbon emissions trading and green financial instruments synergistic? -Comprehensive quantitative research based on content analysis
Source: PLoS One. 2024 Mar 7;19(3):e0298601. doi: 10.1371/journal.pone.0298601 (PMC10919601; doi:10.1371/journal.pone.0298601)
Supplement: S1 Data — (ZIP) [file pone.0298601.s001.zip › Data/地级市Z_F工作报告.zip!/╔╜╬≈/╜·╓╨╩╨/└┤╫╘╣1⁄2╓┌║┼í╛┬φ┐╦╩2╛▌═°í┐_2018.docx]

各位代表：

现在，我代表市人民政府向大会报告工作，请予审议，并请市政协委员和列席人员提出宝贵意见。

一、2017年工作回顾

2017年是晋中发展成果丰硕的一年。在省委、省政府和市委的坚强领导下，市政府坚决贯彻落实习近平新时代中国特色社会主义思想和党的十九大精神，按照省委“一个指引、两手硬”思路要求，紧紧围绕全面挺进全省第一方阵目标，统筹推进促转型、稳增长、惠民生、防风险，各项工作稳中有进，经济社会发展进入新时代。

一年来，我们努力提升经济发展质量效益，综合实力稳步提高。市政府把保持经济稳定运行在合理区间作为贯穿全年的重要工作，坚持经常研究经济重点任务，出台落实国家、省促进资源型经济转型发展、实施创新驱动战略等工作方案，强化经济运行调度，主要指标总体完成目标任务，基本实现全面挺进全省第一方阵的年度预期。全市地区生产总值增长6%，规模以上工业增加值增长4.3%，固定资产投资增长6%，社会消费品零售总额增长6.7%，一般公共预算收入增长17.1%，城乡居民人均可支配收入分别增长6.1%、6.7%。万元地区生产总值二氧化碳排放量、用水量和省下达“大气十条”任务全部完成。干部群众加快发展的信心更强、底气更足。

一年来，我们大力推进转型平台建设，新发展动能加速形成。开辟转型综改主战场，以晋中经济技术开发区为主体，整合扩建山西转型综改示范区晋中开发区，推行“三化三制”改革，172项市级行政管理事项全部授权到位，社会事务剥离、人员分流安置全部完成，开发建设总公司成立运行，晋能光伏二期、新能源商用车等一批重大转型项目落地。以晋中开发区为龙头打造“1+10”联合体，省级开发区新获批3个，通过省级评审5个。启动山西农谷建设，完成规划编制、政策创设、机构运行、投融资平台等四梁八柱的搭建，5条主干路网拉开农谷框架，“一城三园五区”核心支撑要素布局实施，现代农业科技创新中心等6个国家试点项目落地，“一年见雏形”目标基本实现。

一年来，我们着力推动产业升级提质，转型发展呈现强劲态势。制定扶持转型项目和优势企业“2+5”政策体系，激励引导、扶优扶强，全市转型发展趋势性、转折性、结构性变化明显增多。工业经济质效提升，装备制造业增加值增长28.3%，吉利轿车整车生产突破10万辆，东方希望铝形成200万吨氢氧化铝生产能力，安泰集团销售收入突破百亿。新兴产业和创新创业支撑显现，旅游总收入突破800亿，接待游客近8000万人次；奥特莱斯、万达广场等开启多样化新型商业模式，第三产业增加值对GDP增长贡献率达63%；国家玻璃器皿产品质检中心通过验收，新认定高新技术企业16家，省级以上众创空间、科技企业孵化器、重点实验室和工程技术研究中心共新增7个；新登记各类市场主体3.5万户，增长14%。现代农业加快发展，粮食、蔬菜、水果、肉蛋奶产量稳定增长，休闲观光农业快速发展；土地确权基本完成，农村产权流转交易所实现乡镇全覆盖，82%的行政村实现集体经济“破零”。

一年来，我们强力开展城乡建设治理，环境面貌发生明显变化。以环保治理攻坚战为抓手，突出秋冬季大气污染治理特别是燃煤污染控制，全市淘汰燃煤锅炉645台，取缔“散乱污”企业491家，煤改气11.8万户；在市城区105平方公里“禁燃区”开展“燃煤清零”行动，整合热源1200万平方米，新建改造管网700公里，新增集中供热面积700万平方米。市城区百项城建重点工程当年完成投资190亿元，10个城中村整村拆除基本完成。“拆违治乱提质”和农村环境整治攻坚行动持续深入，拆除违建面积419万平方米，腾退土地1.06万亩。新建改建农村公路976.5公里，昔阳、左权实现建制村通硬化路和通客车两个100%，城镇化率达到54.1%。实施244处农村饮水安全工程，巩固提升22万农村饮水安全标准。新造林35万亩，林木绿化率达到35.8%。城乡面貌焕然一新，充满生机。

一年来，我们全力攻坚精准脱贫和民生保障，人民生活持续改善。全市财政投入231亿元用于民生事业，占一般公共预算支出的82.8%。脱贫攻坚扎实推进，又有6.9万贫困人口脱贫、239个贫困村退出，贫困发生率由6.7%降至3.7%，昔阳县通过省贫困县退出检查评估，特别是林业生态建设、文化旅游与脱贫攻坚深度融合为全省趟出新路，“以户为基、五洁净、六要六有”等在全省推广。实施中小学校长职级制和中考制度改革，34个高中校长职级制改革全面完成，4万名初一新生进入“新中考”序列。“国家公共文化服务体系示范区”创建通过文化部中期督查，全市实现市级“五馆一院”、县级“两馆一院”、农村文化活动室、乡镇综合文化站、农村公益电影放映五个全覆盖；成功举办首届平遥国际电影展。新增城乡全民健身活动场所100个，人均体育场地面积1.66平方米。11个县级医疗集团全部组建挂牌，全省首家组建市县乡一体化医疗集团，37家公立医院全部取消药品加成。城乡居民医保制度实现“六统一”，城镇职工和城乡居民医保接入国家异地就医直接结算平台。城镇新增就业4.2万人，城镇登记失业率2.36%。冬季清洁取暖、家庭医生签约、河长制等中央和省市确定的22件实事全面落实。发展成果普惠于民，全市人民的获得感摸得着、看得见，对党的信心、信赖和信仰进一步增强。

一年来，我们竭力建设人民满意的服务型政府，政府职能转型提效。我们严格落实政府系统全面从严治党主体责任，深入推进反腐败斗争。市政府主动向市人大及其常委会报告工作，严格执行人大及其常委会决议决定，主动接受人大、政协监督，共办理人大代表建议252件、政协提案371件。深化“放管服效”改革，在全省实现了“五个率先”，即率先试行企业投资项目承诺制，53个项目进入承诺制流程，加速了项目落地，引领了思想解放；率先制定减证便民“三个清单”；率先推行“准入即营”“多证合一”改革；率先开通“一号通”投诉举报平台；率先成立企业（项目）服务中心，组建百人专职团队常态化驻企帮扶，协调解决问题。“13710”工作制度实现市县乡三级全覆盖。持续改进工作作风，严肃惩治违反中央八项规定精神的问题。干事创业的热情有效释放，团结和谐、风清气正的良好局面正在巩固提升。

一年来，民族、宗教、外事、侨务、港澳、对台、统计、科普、档案、史志、社科研究等工作取得新进展，妇女、儿童、青少年、老龄、残疾人、红十字、慈善等事业取得新进步，气象、地震、治超、援疆等工作取得新成绩，国防后备力量建设、双拥、人民防空工作扎实推进，各项事业生机盎然、蓬勃发展。

各位代表，过去一年的成绩已经融入到晋中大地，铭刻在全市人民心中。这些成绩取得，最根本得益于以习近平同志为核心的党中央正确领导，得益于习近平新时代中国特色社会主义思想的科学指引，也得益于省委、省政府关心关怀，得益于市委统揽全局，得益于市人大、市政协支持监督和全市广大干部群众的团结拼搏。

在此，我代表市人民政府，向全市广大干部群众，向各位代表、各位委员、各民主党派、人民团体和社会各界人士，向驻地人民解放军、武警官兵、公安民警和国家、省驻地单位，向所有关心支持晋中改革发展的朋友们，表示崇高敬意和衷心感谢！

安不忘危，兴不忘忧。我们清醒地认识到，晋中发展仍面临不少困难和挑战，不平衡不充分问题在各领域不同程度存在。特别是实体经济不大不强不优，结构性体制性素质性矛盾远未从根本上解决，科技和人才要素支撑不够；深度贫困地区脱贫攻坚任务艰巨，安全生产形势仍然严峻；政府职能转变还不到位，一些干部服务意识和法治意识还不强、工作作风还不实、担当精神还不够，一些领域不正之风依然存在，等等。我们一定要坚持问题导向，以不畏艰难的勇气、坚韧不拔的意志，尽心竭力做好工作，使人民政府不负人民重托！

二、2018年工作安排

2018年既是贯彻落实党的十九大精神开局之年，也是改革开放40周年，更是决胜全面建成小康社会的关键一年。做好政府工作，要以习近平新时代中国特色社会主义思想为指导，全面贯彻党的十九大精神，坚持稳中求进总基调，紧扣社会主要矛盾变化，按照高质量发展的要求，认真落实省委实现“三大目标”、打好“三大战役”要求，咬定全面挺进全省第一方阵目标不放松，持续实施创新驱动、转型带动、改革推动、项目拉动四大举措，决战转型综改主战场，争创乡村振兴示范市，建设能源革命先行区，打造创新创业新高地，建设富强民主文明和谐美丽新晋中。

决战转型综改主战场。把晋中开发区作为转型综改主战场，全力打造全市开发区“1+10”联合体，形成支撑晋中转型发展和对外开放的重要载体，突出经济属性，强化项目建设，提高首位度、集中度、开放度。

争创乡村振兴示范市。按照产业兴旺、生态宜居、乡风文明、治理有效、生活富裕的总要求，以国家现代农业示范区和山西农谷建设为重点，统筹推进全市农村经济建设、政治建设、文化建设、社会建设、生态文明建设和党的建设，加快推进乡村治理体系和治理能力现代化，促进农业提质增效、农村文明进步、农民增收致富。

建设能源革命先行区。加快传统能源绿色低碳高效开发利用和新能源发展，重点在新能源产业提质、煤炭工业提档升级和扩大先进产能等方面先行先试，着力降低传统能源在能源消费中的比重。

打造创新创业新高地。充分发挥科技创新要素集聚优势，打好政产学研用“组合拳”，唱好创新创业“样板戏”，把资源优势加速转化为创新创业新优势，将晋中打造为山西创新创业的新高地。

综合考虑发展趋势和工作导向，建议全市经济社会发展主要预期指标为：地区生产总值增长6.5%，规模以上工业增加值增长6%，全社会固定资产投资增长9%，社会消费品零售总额增长7%，一般公共预算收入增长8%，城乡居民人均可支配收入分别增长6.5%和6.5%以上，居民消费价格涨幅控制在3%左右，城镇新增就业3.8万人，城镇登记失业率控制在4.2%以内。

约束性指标为：万元地区生产总值能耗下降3.2%、二氧化碳排放量下降3.9%、用水量下降4%。环境质量改善和主要污染物总量减排指标完成省下达任务。农村贫困人口脱贫6.5万人，棚户区住房改造1.48万套。

围绕上述目标，今年重点抓好八方面工作：

（一）扎实推进转型项目建设，增强转型发展供给支撑

把项目建设作为推动转型发展的总抓手，扎实开展转型项目建设年活动，聚力增加有效投资，聚焦优化投资结构，引进和实施一批大项目、好项目，为高质量转型发展夯实基础、增强后劲。

抓实项目前期。突出成长性原则、集聚性效应、有效性导向，积极谋划、策划和包装一批重大项目，提高项目成熟度；建立市县两级项目储备总库和行业子库，储备项目总投资超过2017年固投完成额3倍以上，按季调度、滚动管理；扎实开展“项目集中开工月”活动，4月初续建项目全部复工，新建项目开工40%。

大力招商引资。把招商引资任务量化落实到市县和部门“一把手”头上，加大考核权重，把招商过程、结果作为考核考察干部的重要依据；创新招商方式，完善招商地图，开展模块化招商、产业链招商、以商招商、差异化招商等优质精准招商；选准投资主体，抓好招商项目承接。

完善推进机制。突出转型项目和标杆项目，落实逐月调度、每季市内外企业家项目对接、半年政银企对接等机制，开展“进工地、到一线、解难题”“打击恶意阻工扰工”等专项行动，以政府的担当有为为项目单位排忧解难。用足用活新的占补平衡政策，建立以数量为基础、产能为核心的占补新机制，有效破解用地难题。强化激励导向，对项目推进成效突出的县（区、市），在新增建设用地指标、预算内资金安排、年度目标考核等方面给予激励和倾斜；反之，约谈问责党政主要负责人。

（二）着力构建特色现代产业体系，夯实新旧动能转换基础

坚持存量优化升级与增量培育壮大“双轮驱动”，加快形成符合我市比较优势的现代产业结构。

传统产业要提升层级、循环延伸。用新技术、新模式、新管理等增量促进存量优化升级，更好发挥存量对稳定增长、改善民生的主体作用。煤炭产业，要稳步实施60万吨/年以下矿井减量重组或关闭工作，抓好煤铝伴生资源综合开发和高效循环发展，重点推进灵石煤电铝材一体化产业园建设。焦化产业，要以大机焦改造升级为切入点，打造介休1000万吨以上和灵石500万吨以上新型焦化产业基地。玻璃器皿产业，要积极引进战略投资者，强龙头、组集团，筹办好全国首届玻璃器皿博览交易会，增强行业整体竞争力。纺机产业，要引进中国纺机集团国家级技术中心，推动经纬纺机智能制造园年内开工建设，引领优质配套企业入园，努力建设国内最强纺机基地。

新兴产业要扩大规模、集群集聚。着力打造或培育新能源汽车、现代食品、现代煤化工、现代物流、高端制造、光伏电池、医药、新材料等八个百亿级产业集群、产业基地。新能源汽车产业，重点抓好吉利晋中基地年产15万辆乘用车全产能生产、商用车推广应用与生产，完成GE12电动车技术改造，启动零部件配套企业标准厂房二期建设。光伏电池产业，重点抓好晋能720MW光伏电池及组件项目建设和投产，加快推进上市，打造全省最大的光伏电池及组件生产基地。煤层气产业，要紧紧抓住国家赋予我省煤层气开发重大政策机遇，着力开发榆社—武乡深层煤层气，建设全省大型煤层气产业基地。新能源产业，重点支持寿阳争取成为省级能源革命排头兵试点县。

现代服务业要创新发展、提高水平。文化旅游，突出国家全域旅游示范区创建和太行品牌塑造，全年接待游客突破9000万人次、旅游总收入突破900亿元。支持左权申报创建国家旅游业改革创新先行区，以左权生态文化旅游开发区建设为突破，依托东山五县丰富的山水和红色文化旅游资源，打造山水太行、红色太行、康养太行、动感太行、古韵太行品牌；继续深化旅游景区两权分离和旅游执法体制改革，强化旅游行政执法与司法协调联动；加快旅游公路、厕所、集散服务中心建设；打造一批旅游与康养、低空、体育、户外、医药、艺术融合发展的精品项目。现代物流，重点发挥中鼎物流园区位、硬件和多式联运优势，拓展市场，开展保税业务；推进平遥通用机场建设。

（三）深化重点领域改革创新，不断激发高质量发展的动力活力

着眼晋中新时代发展所需，以更大的力度推进改革、加快创新，特别是发挥市场在资源配置中的决定性作用，着力提高资源配置的效率效能，推动资源向优势企业和产品集中，创新要素自由流动和聚集，通过集中、集聚、集约，形成“火车头”效应，带动、引领、扩大社会创造空间，释放市场活力，使创新成为高质量发展的强大动能。

加快开发区改革创新。引深晋中开发区建设，以高端化、智能化、绿色化为方向，推进金科科技城、百度人工智能产业园、中外合作产业园等重大项目开工建设，打造引领转型的“尖兵部队”。建立规划调控引导、项目落地统筹协调等机制，打造晋中开发区和10县（市）开发区“1+10”联合体，各开发区“三制”改革全部到位，“三化”改革深入推进。加大省级开发区建设和申报力度，支持有实力的民营企业牵头创办省级开发区。

深化金融创新。做强做大地方金融机构，支持晋中银行扩大服务网点和业务规模，加快农信社改制化险扫尾。积极引进省内外金融机构入驻，在省政府《支持现代服务业发展措施（2017年版）》的基础上，结合我市实际进一步提出优惠政策，利用区位优势打造金融洼地，吸引更多银行、证券、基金等机构入驻晋中。创新融资模式，增加市级政策性担保机构资本金、企业应急周转金，增强市级融资平台的发债融资能力。推动企业上市，对新三板挂牌企业市级奖励规模由80万元提高到100万元，对挂牌创新层的再奖励20万元。加强对各类金融机构和互联网金融的监管，继续保持打击非法集资的高压态势，确保不发生区域性金融风险。

强化科技创新和人才支撑。加大科技创新平台建设力度，省级以上重点实验室和工程技术研究中心新增1家、科技企业孵化器新增1家、众创空间新增3家，高新技术企业总数力争突破100家；加快推动科技成果转化，建立完善科技成果信息采集与供需对接体系；积极推动开发区与太原理工大学合作建设应用科学研究院。深入落实《关于深化人才发展体制机制改革的实施意见》，市财政设立不少于3000万元的人才发展专项资金，加大本土人才培养和院士团队引进力度。

推进质量强市建设。着力推动从“有没有”向“好不好”转变，开展质量提升活动，抓好液压铸件降废、H型钢成材率提升等20个质量攻关项目；推进品牌创新战略实施，市财政拿出600万元开展品牌创新成果评价等工作，鼓励企业争创省级、国家级品牌；持续推进精益管理和标准创新分级评定，争创全国质量强市示范城市。

深化国企国资改革。改革国有资本授权经营体制，积极探索有序组建晋中市国有资本投资运营公司，分层推进国有企业混合所有制改革；完成厂办大集体、“三供一业”分离移交等改革年度任务；提高国企国资运行效率，持续优化以经营业绩为导向的考评体系。

（四）大力实施乡村振兴战略，加快农业农村现代化步伐

把实施乡村振兴战略作为做好新时代“三农”工作的总抓手，通过政策和规划引导，制定标准体系，在乡村形成人才、土地、资金、产业汇聚的良性循环，加快实现乡村产业振兴、人才振兴、文化振兴、生态振兴和组织振兴。

强化山西农谷示范引领。聚焦农业科技创新和深化农村改革两大任务，积极争创国家级农业高新技术产业示范区，引领和服务乡村振兴。要在平台打造上取得突破，重点抓好国家科创中心建设，建设功能农业（食品）研究院、农业设施装备研究中心、国家地方联合工程研究中心等创新平台。要在产品开发上取得突破，建设中国食品发酵工业研究院农谷分院，开发3—5个特色功能产品。要在项目建设上取得突破，实施总投资286亿元的63个项目，确保山西农产品国际交易中心如期投入使用，台湾产业园等项目落地开工。要在融合发展上取得突破，推进一二三产、产业化智能化、生产服务三个融合，建成巨鑫、大北农等数字化智能化项目，加快番茄小镇建设。要在农村改革上取得突破，在农村“三变”“三块地”“三位一体”农村合作经济等改革上试点示范。要在园区建设上取得突破，完善“一城三园五区”基础设施，建成国际学术交流中心、标准化厂房等。要在“谷城院”一体化发展上取得突破，设立农谷科技专项基金，探索股权分配、知识价值分享机制。要在品牌建设上取得突破，筹备好中国（阳邑）“三农”改革发展论坛，提升农谷品牌影响力。要在辐射带动上取得突破，以山西农谷农民培训中心为依托，积极申报中国职业农民大学。用好新品种、新技术、新装备，强化与农民利益联结。要在集聚高端人才上取得突破，吸引各类人才在农谷建功立业。

推动产业提质增效。调优区域布局，推进产业园和优势区发展，在每个县（区、市）优先支持建设1个现代农业产业园，扶持壮大10个农产品领军企业、100个骨干企业；开展农业品牌创建年活动，新认证“三品一标”91个，新认定“三品一标”面积31万亩。推进百企千场万户畜牧产业提升工程，积极办好第十三届中国牛业发展大会。加快培育新产业新主体新业态，壮大我市新型现代职业农民队伍，特别是把特色小镇作为产业兴旺的重要抓手，制定特色小镇和特色小城镇发展规划、培育计划、建设标准、扶持政策，年内推进5—10个市级示范项目。

改善农村人居环境。落实国家《农村人居环境整治三年行动方案》，制定出台我市三年行动计划和年度任务。引深“拆违治乱提质”攻坚行动，深入实施美丽宜居示范村“三级联创”活动，创建提升20个以上美丽宜居示范村。抓好农村生态修复治理，全面落实“河长制”，扎实做好汾河核心区干流蓄水运行保障工作。大力推进林业生态建设，完成新造林合格面积35万亩以上，全市林木绿化率达到36.8%以上。深化农村电网精益化升级。加强乡风民风建设，开展“文明村镇”“文明家庭”等创建活动。

深化农业农村改革。全面完成农村承包地确权登记颁证，年内实现全市农村土地承包信息联通共享。加快推进农村集体经营性资产股份合作制改革，扎实做好农村集体建设用地和宅基地使用权确权登记颁证工作。推进农业水价综合改革，各县（区、市）选择1—2个条件较好的乡村开展试点。扩大林权制度改革面。完善粮食收储制度改革，落实粮食安全责任制。加快供销合作社综合改革。

（五）坚决打好打赢精准脱贫攻坚战，补好全面小康短板

全面建成小康社会标志性的指标是农村贫困人口全部脱贫、贫困县全部摘帽。我们要坚决落实习总书记“脱真贫、真脱贫”要求，把提高脱贫质量作为首要任务，把脱贫攻坚与乡村振兴、与脱贫后奔小康统筹结合起来，精准施策、分类指导、合力攻坚，确保6.5万贫困人口脱贫、204个贫困村退出，左权、和顺高标准脱贫摘帽。

深入推进产业扶贫。紧紧围绕“一村一品一主体”和“五有”全覆盖，进一步提升产业、项目对贫困人口的带动力和覆盖面。深化利益联结，积极推广榆社以贫困户为主体发展合作社、左权“身股入社”等经验，增加非失能贫困群众务工参与度，让贫困户有业可从、有企可带、有股可入、有利可获。采取以奖代补等方式充分调动各类新型经营主体的积极性，使其成为带动贫困户增收脱贫的主导力量。

扎实做好易地搬迁。解决好“人、钱、地、房、树、村、稳”等七个问题，重点用好拆迁复垦奖补政策，做好劳务输出、公益岗位与搬迁贫困户的精准对接，加大危房改造力度，完成170个整村搬迁硬任务，1.68万贫困人口搬迁入住，确保搬得出、稳得住、能致富。

强化兜底保障扶贫。持续筑牢因病、因学、因灾“支出型”贫困保障防线，落实好“双签约”“三保险三救助”医保扶贫政策，确保医疗支出报销高比例、全覆盖；深入落实义务教育“两免一补”、雨露计划等教育扶贫政策，高标准实现建档立卡贫困学生资助全覆盖；加快推进养老、医疗、生育、工伤、失业保险对应贫困人口全覆盖，做好建档立卡贫困人口城乡居民养老保险财政代缴，扩大农业保险覆盖面，让贫困群众不因灾减收。

有效激发内生动力。深入开展滴灌式、造血式帮扶，以户为基深入开展“五洁净、六要六有”达标工作，充分激发贫困群众信心、意志和潜能。同步谋划推进脱贫后奔小康工程，发挥左权、昔阳试点示范作用，多渠道持续增收，大力度改善贫困村基础设施，有效提升公共服务水平，为贫困群众与全国人民一道奔小康奠定坚实基础。

（六）持续实施“为民城建”，不断提升城市发展品质

对接太原、晋中、阳泉和山西转型综改示范区“三市一区”发展规划，以共建、共治、共管为切入，以共享、共赢为目标，实施好当年投资357亿元的6大类108项重点工程，不断完善城市功能、提升城市品位，努力让绿色成为城市的主色调、宜居成为城市的影响力、文化成为城市的新名片。

完善公共服务设施。加快补齐公共设施短板，推进残疾人康复中心、消防培训基地、壁画艺术博物馆等建设，确保主体完工，市级“三馆”全面投入使用；优化学校布局，完成广安中学、榆次实验小学等主体工程；提升医疗服务能力，晋中市一院投入使用，完成市中医院门诊综合楼主体等建设；加快城市便民服务设施建设，采取市场化运作方式新建便民市场8处，推进环卫设施提质、环卫停车场建设和固废循环利用。

优化道路交通条件。实施城区环路、主干道、人行过街天桥、小街小巷等25项道路建设工程，加快推进龙城大街东延、综合通道等建设，启动环城东路北延、环城南路西延等项目，进一步完善提升城市内外环快速道路系统；实施安宁街改造、新建路南段改线、广安街东延等主干道建设，进一步打通主干道路断头、疏通梗阻；新建5座人行过街天桥，改造智勇街等15条小街小巷，使市民出行更加便捷、安全。

提升绿化和管理水平。围绕创建国家园林城市，积极启动潇河流域市区段（三期）综合治理，完成顺城公园、学府生态绿地、铁路生态绿色廊道等建设，新增园林绿化面积100万平方米以上，加快形成林在城中、城在林中的绿色格局；全力创建国家卫生城市，市城区和太谷、祁县、平遥、榆社、和顺、寿阳6县年内基本达到创卫标准。完善城乡市容市貌和环境卫生管理考评机制，加快数字化城市管理项目建设，提升城市管理智慧化、精细化水平。

加大住房保障力度。大力发展住房租赁市场特别是长期租赁，通过新建、改建、回购等方式多渠道扩大租赁住房房源；探索完善公租房货币化补贴政策，提高公租房运营保障能力，对8700户住房困难家庭进行廉租住房补贴。市城区重点抓好20个城中村改造，推进蕴华街棚户区、迎宾广场等六个片区改造。

（七）下大力气抓好污染防治，推动环境质量进一步好转

把环境保护作为政治任务和重大民生工程，落实好京津冀及周边地区大气污染防治重点城市责任，坚决打好全市污染防治攻坚战，实行最严格的生态环境保护制度，探索开展市级环保立法，真正将“绿水青山就是金山银山”的理念落到实处。

继续打好蓝天保卫战。一要控煤。扩大市城区“禁燃区”范围，通过整村拆迁改造一批，纳入现有热源集中供热一批，利用现有低排放大燃煤锅炉区域连片供热一批，煤改气兜底一批，确保“禁燃区”内所有散煤用户清洁能源替代全覆盖；同时，对“禁燃区”外影响城区空气质量的重点区域全部实施清洁煤置换。督促推动各县市比照市城区模式，严格落实散煤管控各项措施。二要治污。继续深化“散乱污”企业治理，狠抓小电厂、钢铁、焦化、水泥、碳素等行业企业排污达标。三要降尘。针对建筑施工、房屋拆迁、物料堆场、渣土运输等重点污染源，严格落实抑尘保洁、裸地苫盖等防尘措施。四要管车。市城区组建专业化公司，对所有渣土车实行标准化、规范化统一管理。五要禁烧。重点落实对秸秆、垃圾、烟花爆竹等燃烧燃放的监管责任和管控机制。

深入开展清水、净土攻坚行动。清水重点围绕汾河流域消除劣Ⅴ类水质目标，突出工业废水、城乡生活污水和黑臭水体治理，按时完成工业集聚区集中水处理设施和城市生活污水厂建设，逐步推动废水排放由一级A标准提升到地表水V类标准。净土重点完成土壤环境初步调查，形成污染地块名录，开展典型受污染耕地和污染地块治理修复。

强化能源资源集约节约利用。加大对水泥、钢铁等重点行业、重点企业的节能监察，加强节能技术改造和节能管理，推行能效对标。实施工业能效提升计划，推进余热余压利用等重点节能工程和项目，扶持重大资源综合利用示范项目。落实国土空间规划，合理划定城市开发边界、永久基本农田、生态保护“三条红线”和城镇、农业、生态“三类空间”。

（八）坚持在发展中保障和改善民生，持续增强全市人民的获得感

顺应人民对美好生活的向往，努力办好人民群众的“操心事”“烦心事”，在幼有所育、学有所教、劳有所得、病有所医、老有所养、住有所居、弱有所扶上不断取得新进展，进一步提升人民群众的幸福感。

保障社会安全稳定。牢固树立安全发展理念，扎实开展全市煤矿安全攻坚年活动，实施严厉打击计划外作业、重大事故隐患严查、重大灾害防治、减量重组优化升级、基础提升五个专项行动，强化问责处理，对发生安全事故的煤矿和相关责任人，实行提级处理和问责。落实安全生产巡查制度。统筹加强危险化学品、非煤矿山、建筑施工、道路交通、消防、燃气等行业领域专项整治，确保取得实效。要防范社会治安风险，把扫黑除恶作为重大政治任务，严厉打击各类黑恶势力违法犯罪行为，完善矛盾纠纷多元化解机制，健全公共法律服务体系,为经济社会发展创造安全稳定的环境。

提升公共服务水平。发展公平而有质量的教育，全面推进城乡义务教育一体化改革，促进县域义务教育由基本均衡向优质均衡升级，大力创建义务教育管理标准化学校；持续深化中小学校长职级制改革，全面完成义务教育阶段和学前教育领域的改革，整体启动中小学教师“县管校聘”改革，引深中考制度改革；扩大优质普惠学前教育资源覆盖面，加快城镇幼儿园建设；创新发展职业教育，在市属高职、高专学校新增养老服务、新能源等专业。落实《“健康晋中2030”规划纲要》，扎实推进县乡医疗卫生机构一体化和公立医院改革，进一步完善市、县医疗集团运行机制，深入开展爱国卫生运动，创新食品药品监管，做好健康城镇建设试点工作。推动全民健身和体育惠民，做好二青会承办项目筹备，办好柔力球、滑雪、自行车、垂钓等精品赛事。

推进文化高地建设。加强社会主义精神文明建设，培育和践行社会主义核心价值观。加快现代公共文化服务体系建设，确保国家公共文化服务体系示范区创建通过验收。建设“晋中数字图书馆”“晋中文化云”平台，提高公共文化服务数字化能力和水平。深入实施文化惠民工程，培育新型文化业态。做好文物和非物质文化遗产保护，深入推进国家级晋中文化生态保护实验区建设，办好“2018晋中·故宫文化交流周”。

完善社会保障体系。促进就业高质量，实施全民技能提升工程，落实好高校毕业生就业创业促进计划和培训就业扶贫行动。全面实施全民参保登记覆盖计划，持续推进社保扩面征缴，做好生育和基本医保合并实施试点、机关事业单位养老保险入轨运行、城乡居民统一的基本医疗保险制度、医保跨省异地就医直接结算和医保支付方式改革等工作；提高基本医保和大病保险保障水平，居民基本医保人均财政补助标准再增加40元，达到490元，新增部分一半用于大病保险。全面放开养老服务市场，支持社会资本投资养老服务业，加快县级福利中心、社区居家养老服务中心和老年餐桌建设，开展社区居家“互联网+养老”试点。

同时，要持续发展妇女儿童、老龄、慈善和红十字会等事业。增强做好民族宗教、外事、侨务、对台等工作的意识。支持国防建设，加强双拥工作。做好第四次全国经济普查工作。

各位代表，今年市政府将继续办好群众急需、普遍受益的十件民生实事：实施3630名残疾预防重点干预和残疾儿童抢救性康复项目；对4个贫困县农村妇女进行免费“两癌”检查；为2.72万怀孕妇女提供免费产前筛查与诊断服务；新建70个农村老年人日间照料中心；免费送戏下乡1000场；对5.06万农村转移劳动力、城镇失业人员进行职业技能培训；解决179个村通客车问题，实现行政村全部通客车；无线网络再覆盖75个行政村，实现行政村全覆盖；推进500个以上农村物流节点建设，实现县、乡全覆盖；开展农村厕所无害化改造。

三、建设人民满意的服务型政府

晋中发展进入新时代，对政府自身建设提出了新要求。我们要树牢“四个意识”，增强“四个自信”，自觉维护以习近平同志为核心的党中央权威，坚决落实党中央、省委、市委决策部署，以“朝受命、夕饮冰”的事业心、“昼无为、夜难寐”的责任感，认真履职，努力工作，通过政府优质高效服务推动晋中高质量发展。

坚持依宪依法履职，建设法治政府。全面推进依宪施政、依法行政，对宪法、法律始终保持敬畏之心，把政府所有活动纳入法治化轨道，努力提升政府运用法治思维和方式统筹社会力量、规范社会行为、解决社会矛盾和问题的能力。自觉接受人大依法监督和政协民主监督，认真办理人大代表建议和政协提案，主动接受社会和舆论监督。强化审计监督，加大对审计发现问题整改和督办追责力度，在各级政府及其工作部门、重点直属部门全面推行内审制度。加强政务公开，强化对行政权力的制约监督，确保全市上下在变革中既生机勃勃又井然有序。

推进全面从严治政，建设清廉政府。落实全面从严治党主体责任，切实履行“一岗双责”，营造风清气正的政治生态。深入推进“两学一做”学习教育常态化制度化，扎实开展“不忘初心、牢记使命”主题教育，加强政府系统党风廉政建设和反腐败工作。以永远在路上的恒心和老牛爬坡的韧劲持续转变作风，严格落实中央八项规定及实施细则精神和省市贯彻措施，狠纠“四风”，严肃惩治侵害群众利益的不正之风和腐败问题，整治群众反映“烦死人”“气死人”“累死人”的官僚主义、形式主义问题。领导干部要讲政德，特别要明大德、守公德、严私德，为社会道德建设树立风向标。

加快政府职能转变，建设效能政府。深化政府机构改革，优化机构设置和职能配置，加快推进事业单位分类改革。持续打造“六最”营商环境，引深企业投资项目承诺制改革，形成以“政府服务承诺+企业信用承诺”为核心的企业投资项目审批管理模式；提升便民服务效率，推行“互联网+政务服务”，力争政务大厅一半以上审批事项全程网办、“零站式”服务；梳理编制公共服务事项清单，开展减证便民专项行动，完成市级行政审批事项前置申请、证照年检和政府指定培训清理；在晋中开发区率先开展“证照分离”改革。要以优质的制度供给、服务供给、要素供给，增强发展环境的吸引力和凝聚力。

提升干事创业能力，建设有为政府。职务就是职责，岗位就是战位。要认真落实市委“三个三”工作思路和举措，坚持目标导向、问题导向、结果导向，落实基础工作、重点工作、亮点工作清单，提升政治能力、创新能力、执行能力，扭住关键少数，把以上率下作为提升能力和水平的重要途径，既要率思想、率作风，又要率方法、率能力、率成效，从我做起、从小做起、从实做起，用“真本事”“硬功夫”来担当作为、履职尽责。全面深化、拓展、延伸“13710”信息化督办制度，以 “钉钉子”精神推动各项工作落细落地，努力把晋中的区位优势转化为开放优势、发展优势。完善激励约束、容错纠错机制，旗帜鲜明给积极干事者撑腰鼓劲，对庸政懒政者严肃问责，形成“头雁效应”、激发“群雁”活力，干出实打实的新业绩，干出群众的好口碑。

各位代表，新时代是奋斗者的时代！让我们更加紧密地团结在以习近平同志为核心的党中央周围，在以王成同志为班长的市委坚强领导下，和全市人民一道，以“筚路蓝缕启山林，栉风沐雨砥砺行”的姿态，踏石留印、抓铁有痕，抢抓机遇、奋勇争先，为如期全面建成小康社会、加快实现晋中高质量发展作出新的贡献！
